# Global Gene Expression Analysis Reveals a Link between NDRG1 and Vesicle Transport

**DOI:** 10.1371/journal.pone.0087268

**Published:** 2014-01-31

**Authors:** Hanne A. Askautrud, Elisabet Gjernes, Gjermund Gunnes, Marit Sletten, Douglas T. Ross, Anne Lise Børresen-Dale, Nina Iversen, Michael A. Tranulis, Eirik Frengen

**Affiliations:** 1 Department of Medical Genetics, University of Oslo, Oslo, Norway; 2 Department of Medical Genetics, Oslo University Hospital, Oslo, Norway; 3 Department of Basic Sciences and Aquatic Medicine, Norwegian School of Veterinary Science, Oslo, Norway; 4 Clarient Diagnostic Services, Aliso Viejo, California, United States of America; 5 Department of Genetics, Institute for Cancer Research, Oslo University Hospital Radiumhospitalet, Oslo, Norway; Wayne State University, United States of America

## Abstract

N-myc downstream-regulated gene 1 (*NDRG1*) is induced by cellular stress such as hypoxia and DNA damage, and in humans, germ line mutations cause Charcot-Marie-Tooth disease. However, the cellular roles of NDRG1 are not fully understood. Previously, NDRG1 was shown to mediate doxorubicin resistance under hypoxia, suggesting a role for NDRG1 in cell survival under these conditions. We found decreased apoptosis in doxorubicin-treated cells expressing NDRG1 shRNAs under normoxia, demonstrating a requirement for NDRG1 in apoptosis in breast epithelial cells under normal oxygen pressure. Also, different cellular stress regimens, such as hypoxia and doxorubicin treatment, induced NDRG1 through different stress signalling pathways. We further compared expression profiles in human breast epithelial cells ectopically over-expressing NDRG1 with cells expressing NDRG1 shRNAs in order to identify biological pathways where NDRG1 is involved. The results suggest that NDRG1 may have roles connected to vesicle transport.

## Introduction

N-myc downstream-regulated gene 1 (*NDRG1*) is also known as Drg-1 (differentiation-related gene 1) [1], Cap43 (p43 protein induced by free intracellular Ca^2+^) [2], and RTP (reducing agents and tunicamycin-responsive protein) [3], named by its perceived functions in different *in vitro* assays. The NDRG gene family contains *NDRG1*, *-2*, *-3* and *-4*, which have 57–65% amino acid sequence identity [4]. The tissue-distribution of NDRG1 mRNA is widespread [4], [5], and the NDRG1 amino acid sequence is >80% identical in a variety of species, ranging from amphibians and fish to mammals [6]. However, the physiological functions of NDRG1 remain elusive. *NDRG1* encodes a protein that belongs to the α/β hydrolase superfamily, but lacks an apparent hydrolytic catalytic site [7]. NDRG1 has been shown to exist as a multi-phosphorylated protein, lacks an ER-targeting signal-sequence and has no apparent transmembrane domain [3], [8]. The protein has primarily been detected in the cytoplasm, but is also seen associated with the plasma membrane, nucleus and the inner mitochondrial membrane, depending on the cell type assessed [5].

*NDRG1* missense mutations cause hereditary motor and sensory neuropathy-Lom (HMSNL), an autosomal recessive form of Charcot-Marie-Tooth disease (CMT) also called CMT4D [9]. Patients with CMT4D exhibit early-onset peripheral neuropathy, which progresses in adulthood to severe disability characterized by muscle weakness, sensory loss, and neural deafness [9], [10]. A tumor-promoting role has also been reported for NDRG1 [11], [12], and it has been implicated in metastasis suppression [13]–[15]. Further, NDRG1 has been shown to have a role in myelin sheath maintenance [9], [16], exocytosis in mast cells [8], [17], differentiation [1], [13], [18], and NDRG1 expression is induced by homocysteine [3], androgen [19], as well as in response to various forms of cellular stress such as hypoxia [5], [20], [21], heavy metals [20] or DNA damage [22], [23].

In the present study, we induced DNA damage by doxorubicin treatment of epithelial cell lines expressing NDRG1 at relatively high (SUM102, ME16C2 and HCT116) or low (ZR-75-1 and MCF-7) levels. We found that doxorubicin-induced apoptosis was inhibited in epithelial breast cells expressing shRNAs targeting NDRG1, demonstrating that NDRG1 is required for apoptosis in these cells under normoxic conditions. We also present results showing that NDRG1 is not a regulator of hypoxia-induced gene expression in the epithelial cells studied, and that hypoxia and doxorubicin treatment initiate different NDRG1 responses. Global gene expression analysis of cell lines with manipulated NDRG1 expression demonstrates that NDRG1 influences the expression of genes related to vesicular structures and transport. The roles of NDRG1 in cellular responses to stress, such as hypoxia and apoptosis may converge by NDRG1 exerting its functions at the level of endomembrane structures.

## Results

### NDRG1 does not Regulate Hypoxia-induced Gene Expression

**Figure 1:**
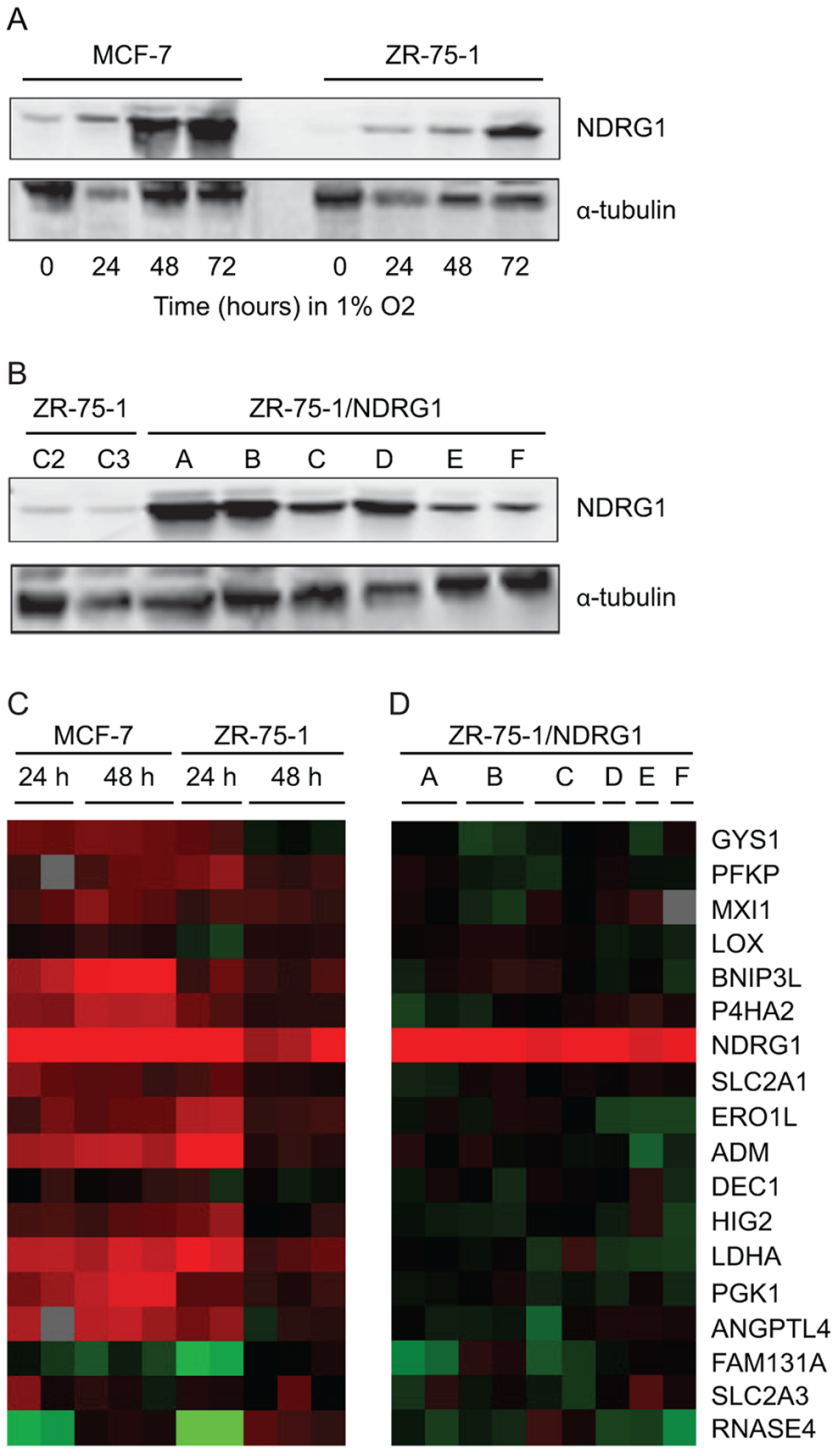
Increased NDRG1 expression due to hypoxia or ectopic expression. (**A**) Increased NDRG1 expression detected by Western blotting in the breast cell lines MCF-7 and ZR-75-1 grown at 1% O_2_ for 24, 48 and 72 hours as indicated, compared to control cells grown at 20% O_2_. (**B**) Western blots document increased NDRG1 expression in cells grown at 20% O_2_. The ZR-75-1 cell populations transduced with the NDRG1-cDNA (six biological parallels indicated A–F), are compared to ZR-75-1 transduced with the vector pSiRPG [27] without insert (C2 and C3). α-tubulin was used as loading control (**A** and **B**). Note that a reduced level of total protein is loaded in the lane containing extract from MCF-7 grown at 1% O_2_ for 24 hours. (**C**) Treeview presentation of the expression of 18 genes in a hypoxia signature gene list [24] in MCF-7 and ZR-75-1 cells grown at 1% O_2_ for 24 or 48 hours as indicated. (**D**) Treeview presentation of the hypoxia signature genes in six ZR-75-1 cell populations ectopically over-expressing NDRG1 (indicated A–F). The red and green colours show increased and reduced expression levels, respectively (**C** and **D**). The colour intensity is from −3 to 3 indicating the magnitude of the fold change in gene expression between the cell population and its control.

We first studied the two luminal breast cancer cell lines, ZR-75-1 and MCF-7, and demonstrated increased NDRG1 expression when these cells were grown at 1% O_2_ for 24 hours (**Figure 1A**). Then we grew both cell lines under hypoxic conditions for 24 or 48 hours and compared the global gene expression profile with cells grown at normoxia. Five microarrays were used per cell line (**Figure 1C**). Sixteen out of 18 genes in a robust hypoxia signature previously described by Chi *et al*. [24] showed increased expression on the microarrays (**Figure 1C**), documenting the hypoxia response in the cells analyzed.

We further investigated whether ectopic NDRG1 expression would result in similar gene expression changes as those detected in cells grown under hypoxic conditions. To facilitate ectopic expression, a construct carrying NDRG1 cDNA (see **Figure S1**) was transduced into ZR-75-1 in parallel experiments resulting in six cell populations, ZR-75-1/NDRG1 A–F, all showing increased NDRG1 expression (**Figure 1B**). Global gene expression profiling was performed using the six ZR-75-1 cell populations ectopically expressing NDRG1 grown in 20% O_2_ (9 microarrays) (see experimental procedures for details). A one class significance analysis of microarrays (SAM) was performed to identify genes with similar expression patterns on these 9 microarrays and the 5 microarrays from ZR-75-1 cells grown under hypoxia. Only genes represented on more than 60% of the arrays were included. The SAM analysis identified only 6 genes in addition to *NDRG1*: *AGMAT*, *FTL*, *KLF12*, *LMNB1*, *SCHIP1*, and *STAT3* (FDR = 0), documenting a limited overlap in the global gene expression response when NDRG1 is induced by hypoxia compared to ectopic NDRG1 over-expression. Furthermore, the only gene in the hypoxia signature showing increased expression in the cells ectopically expressing *NDRG1* was *NDRG1* itself (**Figure 1D**). Thus, ectopic over-expression of NDRG1 does not trigger a gene expression profile resembling the hypoxic response, showing that NDRG1 is not itself a regulator of hypoxia-induced gene expression in the cells analyzed.

### DNA Damage and Hypoxic Growth Induce different NDRG1 Responses in the Cell

**Figure 2:**
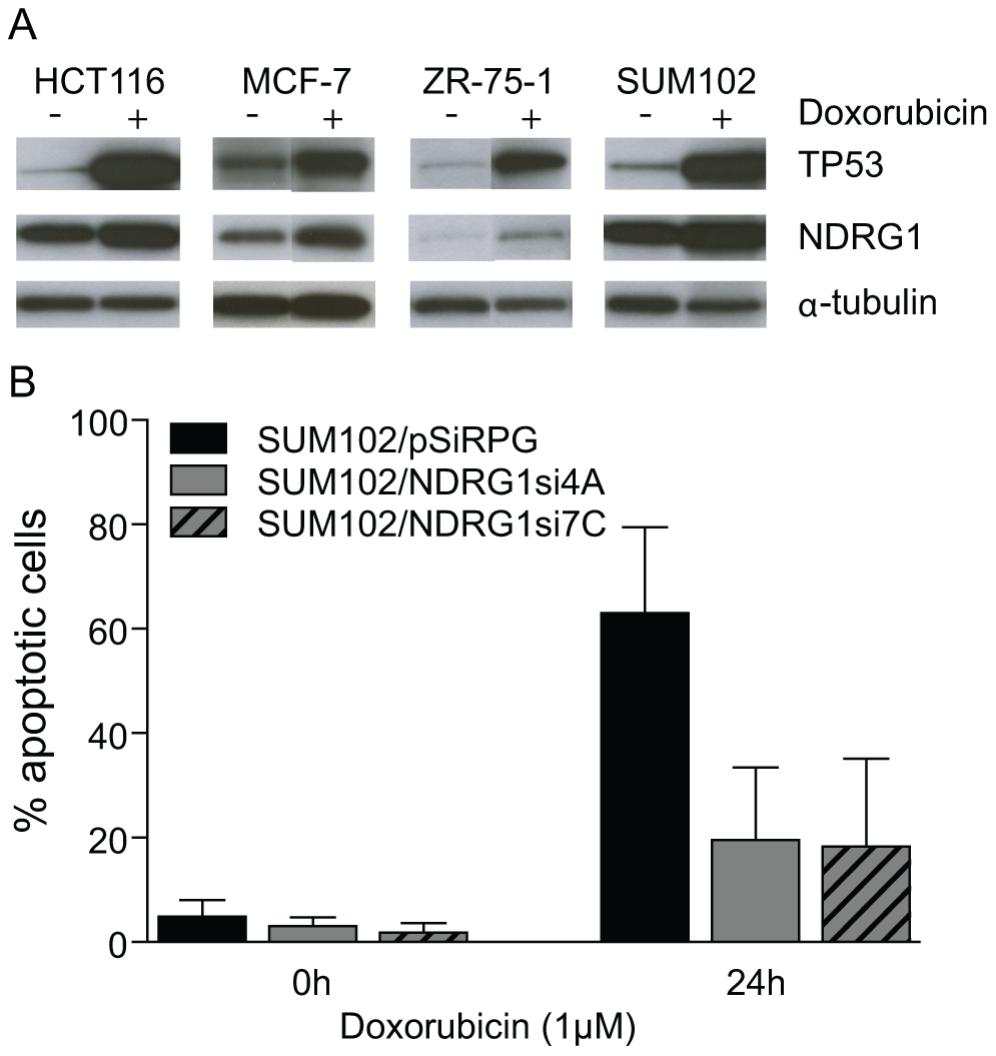
Silencing of NDRG1 inhibits apoptosis in the SUM102 cell line. (**A**) Western blots detect increased TP53 and NDRG1 expression in HCT116, MCF-7, ZR-75-1 and SUM102 cells after treatment with doxorubicin (1 µM) for 24 hours (+) compared to control cells (−). α-tubulin was used as loading control. (**B**) The percentage of apoptotic cells detected by TUNEL assay in the SUM102/pSiRPG, SUM102/NDRG1si4A and SUM102/NDRG1si7A cell lines after treatment with doxorubicin (1 µM) for 24 hours as indicated. Bars are shown with 95% confidence intervals (p<0,02, unpaired two-tailed t-test).

NDRG1 has previously been shown to have a role in TP53-mediated apoptosis in colon and pancreatic cell lines [25], [26]. We detected increased TP53 levels and a distinct increase in NDRG1 levels in a panel of doxorubicin treated cells (**Figure 2A**). Furthermore, we detected a significantly reduced fraction of apoptotic cells when NDRG1 shRNAs [27] were expressed in the immortalized breast cell line SUM102 (**Figure 2B**). This shows that NDRG1 is necessary for doxorubicin-induced apoptosis in SUM102 under normoxic conditions.

**Figure 3:**
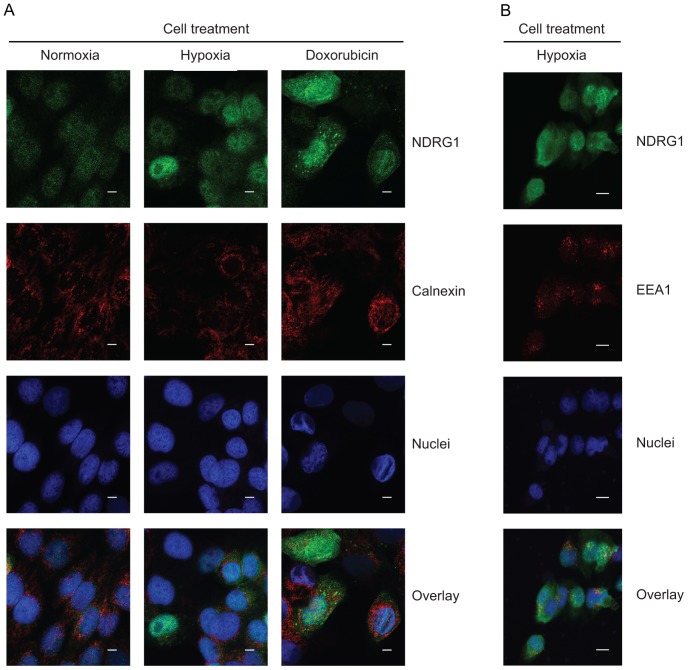
Immunofluorescence staining of SUM102 cell cultures. Colors were applied artificially for graphical purposes. (**A**) NDRG1 (green) and calnexin (red) in untreated, hypoxia and doxorubicin treated cells. Bar: 5 µm. (**B**) NDRG1 (green) and EEA1 (red) in hypoxia treated cells. Bar: 10 µm.

We then treated SUM102 cells with doxorubicin and in parallel grew cells in hypoxic conditions, and used immunofluorescence to detect NDRG1 (**Figure 3A**). Cells grown at 1% O_2_ show increased NDRG1 levels in the cytoplasm, whereas doxorubicin treatment resulted in increased NDRG1 levels with a coarsely granulated cytoplasmic staining pattern (**Figure** **3A**). Staining with markers for ER (calnexin) and early endosomes (EEA1) did not show co localization with NDRG1 in SUM102 under the conditions tested (**Figure 3A,B**). Furthermore, we did not observe any obvious morphological differences in the SUM102 cells with or without NDRG1shRNA expression under the conditions tested (results not shown). In summary, our results indicate that hypoxia and doxorubicin treatments initiate different NDRG1 responses in SUM102 cells.

### Global Gene Expression Analysis Indicate that NDRG1 Function is Connected to Endomembrane Structures and Vesicular Transport

**Figure 4:**
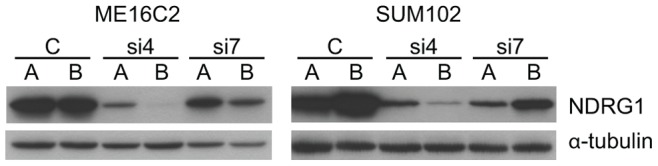
Knock down of NDRG1 expression by shRNAs. Western blots detect reduced NDRG1 expression in the breast cell lines ME16C2 and SUM102 expressing the shRNAs NDRG1si4 (si4) and NDRG1si7 (si7), transduced in parallel experiments (A and B) compared to control cells (C) transduced with the empty vector pSiRPG [27]. α-tubulin was used as loading control.

**Figure 5:**
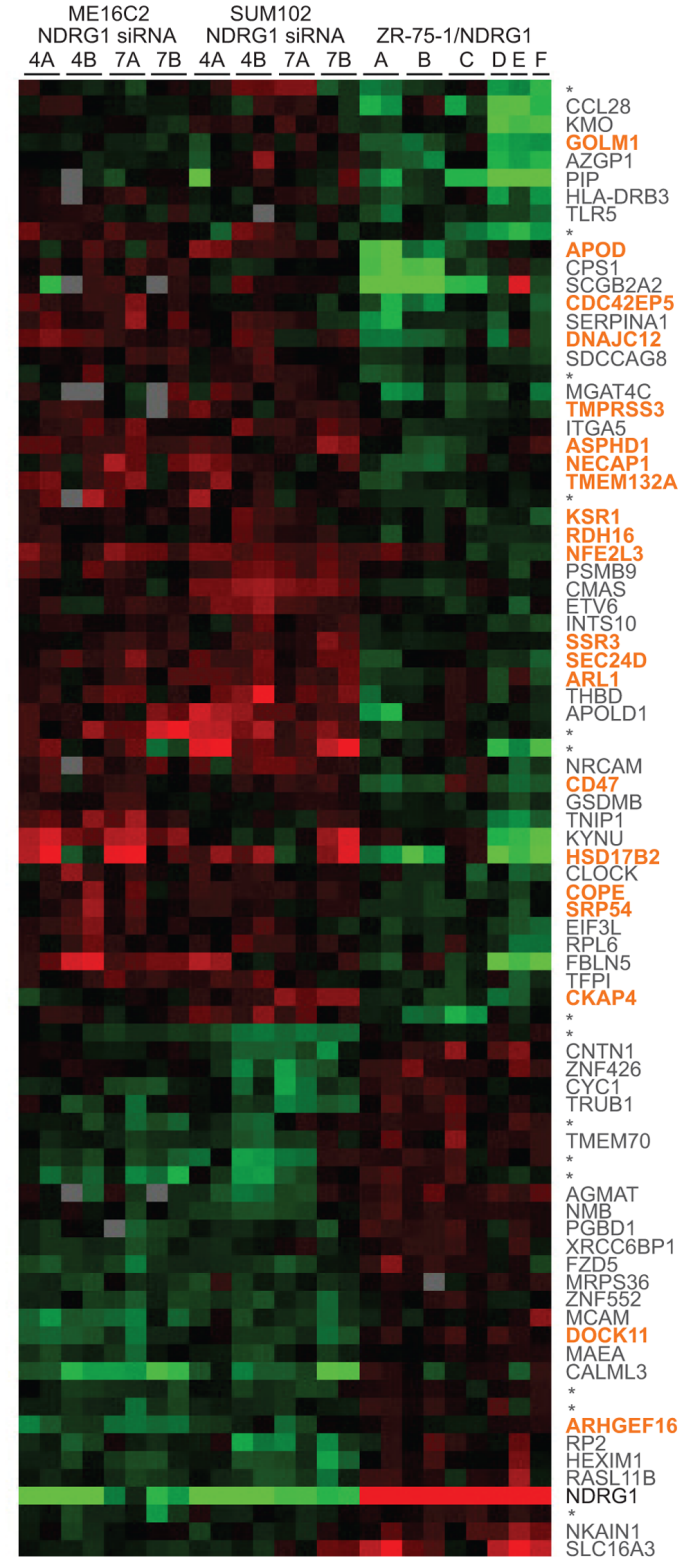
Gene expression profile of cells with manipulated NDRG1 expression. Treeview presentation of the 83 probes representing genes found to be significantly differently expressed in SUM102 and ME16C2 cells expressing NDRG1 shRNAs when compared to ZR-75-1 with ectopic NDRG1 over-expression. The results show a hierarchical clustering of the gene expression pattern, while the arrays are sorted according to cell populations. Genes with no available functional information (14 genes) are indicated with (*). Genes annotated in orange encode proteins that are linked to ER, Golgi, endosomes, as well as genes connected to vesicle transport. The expression of NDRG1 (black) confirms the ectopic over-expression in ZR-75-1 and shRNA-based down-regulation in SUM102 and ME16C2 cells. The red and green colours show over-expression and down-regulation respectively. The colour intensity varies between −2 and 2 indicating the magnitude of the fold change in gene expression between the cell population and its control.

To investigate the global gene expression changes caused by manipulated NDRG1 expression, we focused on the breast epithelial cell lines SUM102 and ME16C2, because both have high endogenous NDRG1 expression (**Figure 4**). Two NDRG1 shRNA constructs, NDRG1si4 and NDRG1si7 [27], were transduced into these cell lines in two parallel experiments resulting in a total of eight cell populations with reduced NDRG1 expression: SUM102/NDRG1si4A and 4B, SUM102/NDRG1si7A and 7B, ME16C2/NDRG1si4A and 4B, and ME16C2/NDRG1si7A and 7B (**Figure 4**). Global gene expression profiling was performed and a two class SAM analysis was used to identify genes showing different expression in SUM102 and ME16C2 with NDRG1 knock-down (16 microarrays) compared to ZR-75-1 ectopically over-expressing NDRG1 (9 microarrays). The SAM delta value was adjusted to obtain the largest gene list that gave a false discovery rate of less than 5% identifying 103 probes representing 83 genes (FDR = 3.5%) (**table S1**). The remaining probes represented control oligos, duplicated oligos and multiple oligos targeting the same gene. Clustering of the resulting 83 genes identified across the different cell populations are visualized in **Figure 5**.

Functional information was available for 69 of the 83 genes in the SAM-derived gene list and these were used to search for enriched gene ontology terms by Gominer [28]. The cellular compartments identified in the Gominer analysis using a cutoff for significant GO categories at p≤0.005, are listed in **table S2**; vesicle coat (3 genes), organelle membrane (13 genes), extracellular space (10 genes), membrane coat (3 genes), integral to organelle membrane (4 genes), and endoplasmic reticulum (11 genes), in total adding up to 26 of the 69 genes (as several of the genes are present in more than one category). Motivated by the Gominer results, we further reviewed the literature regarding the remaining genes in the SAM-derived gene list. The products from an additional 8 genes were found to be involved in ER to Golgi, Golgi to ER or endosomal transport, and 2 were found to be involved in endocytosis and exocytosis. In summary, of the 69 genes with functional information in the SAM-derived gene list, a total of 21 genes were shown to be involved in processes connected to vesicle transport (indicated in orange in **Figure 5**). Nineteen of these 21 genes showed an opposite expression pattern compared to NDRG1; i.e. they were up-regulated in the cell populations where the NDRG1 level was reduced by shRNAs, and down-regulated in ZR-75-1 with ectopic NDRG1 expression (indicated in orange in **Figure 5**). These results suggest that induced changes in NDRG1 expression affect the expression of genes involved in vesicle transport.

## Discussion

### NDRG1 Exerts its Effect through Different Stress-signalling Pathways

Hypoxic stress has been shown to induce NDRG1 expression in a variety of cells [29], but NDRG1’s roles during hypoxia are not fully understood. We identified a similar expression pattern of only seven genes, including *NDRG1,* when comparing the global gene expression profile in ZR-75-1 cells grown under hypoxia with cells ectopically over-expressing NDRG1. Thus, NDRG1 does not act as a sensor of intracellular oxygen tension. The elevated NDRG1 expression is rather a secondary effect of hypoxic stress.

NDRG1 is induced both by ectopically expressed and endogenously increased TP53, the *NDRG1* promoter contains a putative TP53 binding site [25], [30], and NDRG1 has been shown to be necessary for TP53-dependent apoptosis in colon and pancreatic cells [25], [26]. In line with this, we detected decreased apoptosis in doxorubicin-treated breast epithelial cells expressing NDRG1 shRNAs under normoxia. Other studies show no correlation between NDRG1 expression and apoptosis despite the up-regulation of TP53 [15], [21], [30], [31], suggesting different responses depending on the cell types and conditions studied. Jung *et al*. [32] demonstrated that hypoxia-induced NDRG1 expression can mediate doxorubicin resistance, suggesting that NDRG1 might increase cellular survival under hypoxia. We observed strongly elevated NDRG1 levels under hypoxia and in cells subjected to acute DNA-damage by doxorubicin treatment. Under hypoxia we detected an even cytoplasmic NDRG1 signal, whereas doxorubicin treatment resulted in a granulated signal. NDRG1 did not co-localize with ER or early endosomes under the conditions tested. Our results are consistent with the report by Shi *et al*. [33], who detected NDRG1 at the cell membrane and the cytoplasmic network closely associated with the ER in trophoblasts under hypoxia. Our observations are in agreement with NDRG1 exerting its functions through different stress-signalling pathways, resulting in increased cell survival under hypoxia, while being permissive for doxorubicin-induced apoptosis under normoxic conditions.

### Global Gene Expression Analysis Links NDRG1 to Endomembranes and Vesicular Transport

Several of the genes identified in the current global expression profiling encode proteins located in the ER (*NFE2L3, ASPHD1, CKAP4, HSD17B2, TMPRSS3, KSR1* and *RDH16*), proteins required for endosome to Golgi trafficking (*ARL1*), for transport through Golgi (*GOLM1*), for the retrograde Golgi-to-ER transport (*COPE*), and for vesicle budding from the ER (*SEC24D*). The microarray results also revealed genes encoding proteins involved in the targeting of proteins to the ER (*SRP54*), ER membrane translocation (*SSR3*), and proteins needed for chaperone function (*DNAJC12* and *TMEM132A*). Previous studies have suggested that NDRG1 might be a client protein of the chaperone HSP90, or that NDRG1 is a chaperone protein itself [19]. In our microarray experiments we detected *CD47* and *NECAP1*, the latter inhibits endocytosis when over-expressed [34], and CD47 trigger exocytosis when activated [35], [36]. In total, 21 of the 69 differently expressed genes detected in the gene expression profiling in cells with manipulated NDRG1 expression encode proteins that are linked to ER, Golgi, endosomes or vesicular transport between these compartments (indicated in orange in **Figure 5**). Nineteen of these 21 genes are up-regulated in cell populations with NDRG1 knock down, and down-regulated in cell populations with ectopic over-expression of NDRG1, which indicates that NDRG1 facilitates a down-regulation of several genes encoding proteins in the ER to endosome axis. In stressful situations with lack of energy, such as under hypoxia or during ER-stress, a down-regulation of some activities in the secretory and/or endocytic routes of the cell might conserve energy and thus contribute to restore cellular homoeostasis. It is tempting to speculate that NDRG1 might increase cell survival during hypoxia through such a mechanism.

CDC42 belongs to the Rho family of GTPases and influences vesicle trafficking at many levels, anterograde ER to Golgi, post-Golgi, exocytosis as well as endocytosis and further retrograde transport via interaction with Wiskott-Aldrich syndrome protein (N-WASP) and the Arp2/3 complex leading to altered actin dynamics [37]–[39]. Three of the genes detected in our microarray analysis, *CDC42EP*, *DOCK11* and *ARHGEF16* encode proteins that modulate the activity of CDC42: While CDC42EP negatively regulates CDC42 function, both DOCK11 and ARHGEF16 specifically activate CDC42 [40]–[43]. In cells over-expressing NDRG1, the levels of *CDC42EP*, *DOCK11* and *ARHGEF1* detected on the micro arrays are shifted in a direction that is in agreement with an induction of increased CDC42 activity, conceivably resulting in increased vesicle trafficking. Thus, NDRG1 may indirectly play a role in vesicle trafficking and in maintenance of cellular polarity, by stimulating CDC42. Interestingly, hyperactivation of CDC42 can contribute to cellular transformation and tumor invasion and metastasis, through generation of plasma membrane protrusions, so-called invadopodia [39]. Further investigations are needed to clarify the putative interplay between NDRG1 and CDC42 signalling pathways.

In humans, Alaskan Malamute and Greyhound, germline *NDRG1* mutations cause the demyelinating disorder CMT4D [9], [44] and *Ndrg1* deficient mice show defects connected to myelin sheath maintenance [16], [45]. Generally, many genes affected in Charcot-Marie-Tooth disease include proteins involved in regulation of endocytosis and vesicle transport [46]–[48]. In a study of an early onset demyelinating disease, classified as CMT4H in one Lebanese and one Algerian family, Delague and colleagues [49] identified novel mutations in a protein directly influencing the activity of CDC42. The protein, known as FRABIN (FGD1-related F-actin binding protein) is a specific regulator of CDC42 activity and it was postulated that the mutations led to loss of CDC42 activity, leading to reduced F-actin positive filipodia protrusions in cell cultural experiments. Whether CDC42 activity is compromised also in CMT4D, due to NDRG1 inactivation, is presently unknown, and might thus provide a starting-point for further studies into the molecular pathogenesis of CMT4D.

Recently, cell death signals have been shown to induce trafficking of recycling endosomes through a pathway involving CDC42 [50], and CDC42 was shown to be involved in FAS-enhanced membrane trafficking [51]. Also, TP53 can induce apoptosis through death receptors by increased expression of FAS [52]. NDRG2 silencing has been shown to inhibit TP53-mediated apoptosis [53], and inactivation of NDRG2 may elicit resistance against FAS-mediated cell death [54]. Our microarray results may indicate an involvement of NDRG1 in trafficking of recycling endosomes. Additional research is required to explore if increased NDRG1 levels could result in TP53-mediated apoptosis through increased vesicle formation, internalization and recycling of FAS through activation of CDC42. Furthermore, the reported tumor-promoting role of NDRG1 [11], [12] and the role in metastasis suppression [13]–[15], which are apparently contradictory may be explained by involvement of NDRG1 both in cell survival under hypoxia and in doxorubicin-induced apoptosis under normoxia. In summary, the pleotropic roles of NDRG1 reported in apoptosis, cell survival, myelin sheath maintenance and enhanced exocytosis in mast cells, and in the cellular responses to hypoxia, heavy metals, and androgenes may converge by NDRG1 influencing vesicular trafficking.

## Materials and Methods

### Cell Culture and Generation of Stable Cell Populations

The breast epithelial cell lines ME16C2 [55], and SUM102, a gift from Steve Ethier of Wayne State University (Asterand, MI, USA), and their derivatives were grown in TCF-EGM developed by the Tissue Culture Facility at the University of North Carolina at Chapel Hill (http://www.unclineberger.org/tcf/index.asp). The luminal breast cancer cell lines ZR-75-1 (CRL 1500; ATTC, Manassas, VA, USA) and MCF-7 (HTB-22; ATTC) and their derivatives were grown in RPMI1640 with phenol red (Lonza, Walkersville, MD, USA) supplemented with 2 mM L-glutamine (Invitrogen, Carlsbad, CA, USA) and 10% FBS (Invitrogen). HCT116 (CCL-247; ATTC) was grown in McCoy’s 5a medium (Lonza) supplemented with 10% FBS. For the hypoxia experiments cells were grown in a hypoxic chamber, MIC-101, (Billups-Rothenberg, Del Mar, CA, USA) at 1% O_2_.

Transductions were performed as previously described [27]. Stably transduced cell populations were established by selection with puromycin (2 µg/mL; Invitrogen) for 2–5 weeks. All cell populations were analyzed by Western blotting for expression of NDRG1. Cell populations transduced with the vector pSiRPG [27] without insert were also created as controls.

### Western Blotting

Cells harvested in PBS were lysed in M-PER (Thermo Fisher Scientific, Rockford, IL, USA) containing Halt Protease Inhibitor and 5 mM EDTA (Thermo Fisher Scientific). Protein concentrations were quantified using a NanoDrop® ND-1000 Spectrophotometer (Thermo Scientific, Wilmington, DE, USA) or with a dye-binding assay (Bio-Rad, Hercules, CA, USA. Protein electrophoresis, western blotting and immunodetection were performed as previously described [27]. The blots were incubated over night with the NDRG1 antibody (diluted 1∶1000; Clarient, Aliso Viejo, CA, USA). A monoclonal α-tubulin antibody (diluted 1∶1000; SIGMA Aldrich, St. Louis, MO, USA) was used as loading control. Secondary antibody was horseradish peroxidase-conjugated anti-rabbit or anti-mouse, respectively (diluted 1∶5000; GE Healthcare, Buckinghamshire, United Kingdom), which was detected by enhanced chemiluminescence (ECL Plus, GE Healthcare). Blots in Figure 1 were visualized with alkaline phosphatase-conjugated secondary antibodies (Bio-Rad) and fluorescence was recorded with a variable mode imager (Typhoon 9200, GE Healthcare).

### Global Gene Expression Analysis

Total RNA was isolated from all cell populations using RNAeasy (Qiagen, Hilden, Germany). Cells grown under hypoxic conditions (24 h and 48 h) and control cells incubated at normoxia were harvested in lysis buffer at the same time points. RNA was labeled with Cy3- or Cy5-CTP [56]. To minimize the dye effects, three of the cell populations, ZR-75-1/NDRG1 A/B/C, were labeled with both Cy3- and Cy5-CTP and mixed with the control RNA labeled with the opposite dye. The three remaining ZR-75-1 populations were labeled once, but population E was labeled with the opposite dyes compared to population D and F. For ZR-75-1 and MCF-7 grown under hypoxia, all parallels were labeled once, but two of the parallels harvested after 48 hours were labeled with the opposite dyes compared to the other samples. For the cell populations expressing shRNAs, each sample was labeled with both dyes in a dye flip set-up. Samples were hybridized to either Human 1A (V2) Oligo Microarrays (G4110B) or Human Whole Genome Oligo Microarrays (G4112A) (Agilent, Santa Clara, CA, USA). Scanning were performed using a GenePix 4000B scanner (Molecular Devices, Sunnyvale, CA, USA) adjusting the PMT settings for each channel and each array so that the count ratio was close to 1 and the intensities of the spots did not exceed the maximum detection limit. Image analysis was performed using GenePix Pro 6 (Molecular Devices). Loess Normalization was performed to adjust microarray data for variation between the samples.

All microarray data reported in this paper is MIAME compliant and the raw data has been deposited in the GEO database (GEO accession number GSE33439).

### Statistical Analysis

Both one class and two class SAM (SAM version 3.02) was performed using 100 permutations and 10 k-nearest neighbors [57]). The SAM delta value was adjusted to give a false discovery rate (FDR) less than 5%. The SAM derived gene lists were clustered using the average linkage hierarchical cluster analysis in the Cluster program [58] sorted on samples, and the data were visualized in Treeview [59]. The SAM derived gene list was analyzed for the cellular component gene ontology using High-Throughput GoMiner [60]. All genes represented on the microarray were entered as a background file. A one sided p-value was calculated for each category based on a Fishers exact test. The threshold of significance for a category was defined as p≤0.05.

### Measuring Apoptosis by TUNEL Assay

SUM102 seeded at 200.000 cells per cm^2^ adhered for 24 h before treatment with doxorubicin (1 µM; Sigma-Aldrich) for 24 hours. Duplicates were harvested and washed twice with PBS and fixed in 4% paraformaldehyde in PBS for 15 min at 4°C. Cells were pelleted by centrifugation and permeabilized in pure methanol for at least 20 min at −20°C. The cells were washed twice with PBS and apoptotic cells detected by terminal deoxynucleotidyl transferase mediated dUTP end-labeling (TUNEL) assay using the *In situ* cell death detection kit, Fluorescein (Roche Applied Science, Mannheim, Germany). The cells were incubated for 60 min at 37°C with TdT and fluorescein, centrifuged and stained with Hoechst 33258 (2 µg/ml; Invitrogen) for 15 min on ice. The cells were filtered through a nylon mesh and 10000 events were analyzed for each sample using FACS DIVa Vantage Cell Sorter (Becton Dickinson, Heidelberg, Germany) and data collection carried out using the CellQuest 3.3 software (BD Biosciences, Franklin Lakes, NJ, USA).

### Immunofluorescence

SUM102 cells were plated on glass chambered slides and grown to 60–80% confluence. The cells were fixed in 2% paraformaldehyde in PBS, washed and treated with 0.2% Triton X-100 in PBS for permeabilization. After blocking (2% BSA, 0.2% Tween 20, 7% Glycerol, 2% Goat serum, in PBS), cells were incubated with NDRG1 antibody (rabbit, diluted 1∶200; Clarient) or isotype control antibodies (Invitrogen) over night. Secondary antibody was Alexa-405 anti-rabbit (diluted 1∶250; Invitrogen A31556). Early endosomes were marked with the EEA1- antibody (mouse monoclonal IgG1 diluted 1∶1000; Abcam ab70521), and detected with secondary anti-mouse IgG1 antibody Alexa-633 (diluted1∶1000; Invitrogen A21126). Endoplasmic reticulum was marked with an anti-calnexin antibody (mouse monoclonal IgG1, diluted 1∶1000; Abcam ab31290) and secondary anti-mouse IgG1 antibody Alexa-488 (diluted 1∶200; Invitrogen). Propidiumiodide (PI), was used in a 1∶10 dilution for nuclear contrast. Images were acquired using a Zeiss LSM 710 confocal laser scanning microsope (Carl Zeiss, Jena, Germany). Lasers and fluorescent filters were optimized for 405 nm (NDRG1), 488 nm (calnexin), 560 nm (PI), and 633 nm (EEA1) excitation maxima. To facilitate signal interpretation, pseudocolors were applied digitally, using the Zen Lite 2011 software package (Carl Zeiss, Jena, Germany). The aquired images were assembled in Adobe Photoshop Elements 10 (Adobe Systems Inc., San Jose, California, USA).

### Accession Number

The micro array results reported in this paper have been assigned the GEO accession number GSE33439.

## Supporting Information

Figure S1 The expression vector pRTRex. The vector pRTRex is compatible with the Gateway system where the cDNA replaces the fragment between the attR-sites using the LR clonase (Invitrogen, CA, USA). The phosphoglycerate kinase (PGK) and Cytomegalovirus (CMV) promoters are shown with open arrows. The puromycin resistance gene (PuroR) and the Long terminal repeats (LTR) from murine stem cell virus are shown as black arrows. The vector is based on a pUC vector backbone for high copy number propagation in *E. coli.* The map was constructed by using Vector NTI (Invitrogen). The pRTRex vector was constructed as follows; the plasmid pSUPER.retro.Puro (OligoEngine, WA, USA) was digested with *Eco*RI and *Xho*I, and pT-Rex-DEST30 (Invitrogen) was digested with *Nhe*I and *Pci*I. The 5′-overhangs created by these enzymes were blunt ended with the Klenow fragment of DNA polymerase 1 (New England Biolabs (NEB), MA, USA). The resulting two fragments were religated, transformed into into *E.coli* DH5α and colonies were analyzed by restriction mapping and by sequencing over the cloning junctions using the primers pTRexS1 (CCC CTT GAA CCT CCT CGT TC) and pTRexS2 (GCC AGA GGC CAC TTG TGT AG). A clone with the correct sequence was named pRTRex. This Gateway compatible expression vector is available upon request. A construct named pRTRex-NDRG1 contains the NDRG1 cDNA insert (MGC-11293, ATCC-LGC), which was transferred via pDONR201 (Invitrogen) using the BP clonase, and further transferred to pRTRex using the LR clonase (Invitrogen).
(TIF)

Table S1 Expression values for genes showing significantly different expression between cells with NDRG1 knock down versus ectopic over-expression of NDRG1. Columns A–E show symbol, name, Genbank Accession number, UniGene ID and Agilent ID, respectively, for each of the genes. Columns F-AD show the relative expression values in the cell populations indicated (header row) compared to the expression in its respective control. Log_2_ of the ratios of the colour intensity in the cells with altered NDRG1 expression versus the controls are given.
(XLS)

Table S2 Enriched gene ontology terms indentified by GoMiner analysis. Genes are listed alphabetically in the first column of the table. The header row gives the name of the GO category, sorted by increasing p-values. Categories with p-value above 0.005 are excluded. (+) indicates association with a GO category.
(XLS)
