# Supplementary figures and images for: Global Gene Expression Analysis Reveals a Link between NDRG1 and Vesicle Transport
Source: PLoS One. 2014 Jan 31;9(1):e87268. doi: 10.1371/journal.pone.0087268 (PMC3909102; doi:10.1371/journal.pone.0087268)

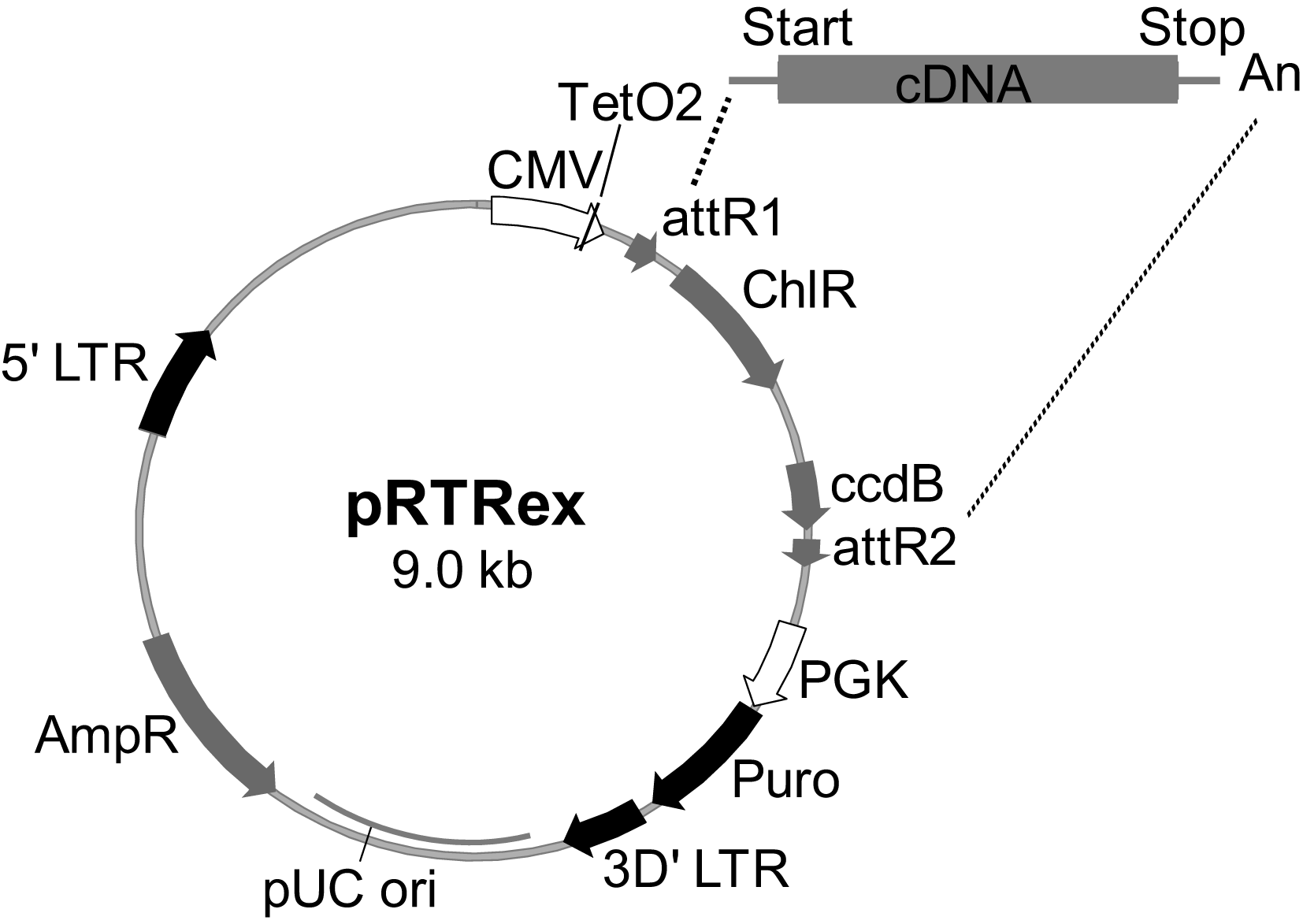

Supplement: Figure S1 — The expression vector pRTRex. The vector pRTRex is compatible with the Gateway system where the cDNA replaces the fragment between the attR-sites using the LR clonase (Invitrogen, CA, USA). The phosphoglycerate kinase (PGK) and Cytomegalovirus (CMV) promoters are shown with open arrows. The puromycin resistance gene (PuroR) and the Long terminal repeats (LTR) from murine stem cell virus are shown as black arrows. The vector is based on a pUC vector backbone for high copy number propagation in E. coli. The map was constructed by using Vector NTI (Invitrogen). The pRTRex vector was constructed as follows; the plasmid pSUPER.retro.Puro (OligoEngine, WA, USA) was digested with EcoRI and XhoI, and pT-Rex-DEST30 (Invitrogen) was digested with NheI and PciI. The 5′-overhangs created by these enzymes were blunt ended with the Klenow fragment of DNA polymerase 1 (New England Biolabs (NEB), MA, USA). The resulting two fragments were religated, transformed into into E.coli DH5α and colonies were analyzed by restriction mapping and by sequencing over the cloning junctions using the primers pTRexS1 (CCC CTT GAA CCT CCT CGT TC) and pTRexS2 (GCC AGA GGC CAC TTG TGT AG). A clone with the correct sequence was named pRTRex. This Gateway compatible expression vector is available upon request. A construct named pRTRex-NDRG1 contains the NDRG1 cDNA insert (MGC-11293, ATCC-LGC), which was transferred via pDONR201 (Invitrogen) using the BP clonase, and further transferred to pRTRex using the LR clonase (Invitrogen). (TIF) [file pone.0087268.s001.tif]
